# Predation of the endangered Ae'o (Hawaiian Stilt) by a native raptor, the Pueo (Hawaiian Short‐eared Owl) on the island of O'ahu, Hawai'i, USA

**DOI:** 10.1002/ece3.10844

**Published:** 2024-01-15

**Authors:** Marie‐Sophie Garcia‐Heras, Jessica L. Idle, Olivia Wang, Kristen C. Harmon, Chad J. Wilhite, Kaleiheana‐a‐Pōhaku Stormcrow, Wade H. Naguwa, Lesley N. Davidson, Lauren S. Katayama, Melissa R. Price

**Affiliations:** ^1^ Department of Natural Resources and Environmental Management University of Hawai'i at Mānoa Honolulu Hawaii USA

**Keywords:** conservation management, Hawaiian islands, predation, predator–prey interactions, raptors, waterbirds

## Abstract

While the impact of introduced predators is a widely acknowledged issue and key component of conservation considerations for endemic waterbird populations in the Hawaiian Islands, the impact of native predators on endemic, endangered waterbirds is not as frequently discussed or factored into recovery models. The Pueo (Hawaiian Short‐eared Owl; *Asio flammeus sandwichensis*) is a subspecies of Short‐eared Owl endemic to the Hawaiian Islands and is State‐listed as Endangered on the island of O'ahu. The Ae'o (Hawaiian Stilt; *Himantopus mexicanus knudensi*) is a subspecies of the Black‐necked Stilt endemic to Hawai'i and is federally listed as Endangered throughout its range. A variety of non‐native predators are confirmed to consume Ae'o eggs, chicks, and adults, including invasive mammals (e.g., feral cats), birds (e.g., Barn Owls), and amphibians (e.g., bullfrogs). While predation by native predators was suspected, there are no cases documented in the literature to date describing Pueo preying upon Ae'o. Here, we describe four events that provide evidence of Pueo predating Ae'o during the 2019–2021 breeding seasons in a wetland area on the island of O'ahu: (1) confirmed Pueo predating an Ae'o chick, (2) a suspected predation attempt of a Pueo chasing adult Ae'o, and (3) two suspected predation events based on (a) 10 adult‐sized Ae'o carcasses and remains found near an active Pueo nest and (b) game camera photos of Pueo visiting two Ae'o nests. To our knowledge, these novel observations are the first published accounts of predator–prey interactions between these two subspecies.

## Graphical abstract

The Pueo (Hawaiian Short‐eared Owl; *Asio flammeus sandwichensis*) is a subspecies of Short‐eared Owl endemic to the Hawaiian Islands and is considered endangered on the island of O'ahu, while the Ae'o (Hawaiian Stilt; *Himantopus mexicanus knudensi*) is an endangered subspecies of the Black‐necked Stilt (*Himantopus mexicanus*) that is native to Hawai'i. In this note, we describe four different events that provide evidence of Pueo preying upon Ae'o during the 2019–2021 breeding seasons in a wetland area on the island of O'ahu. To our knowledge, these novel observations are the first published accounts of predator–prey interactions between these two globally distributed species.

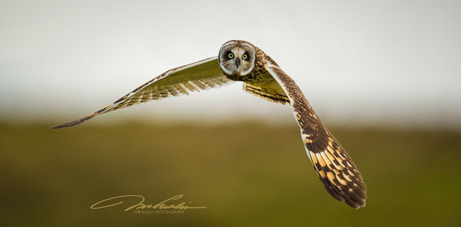

## 1 INTRODUCTION

**FIGURE 1:**
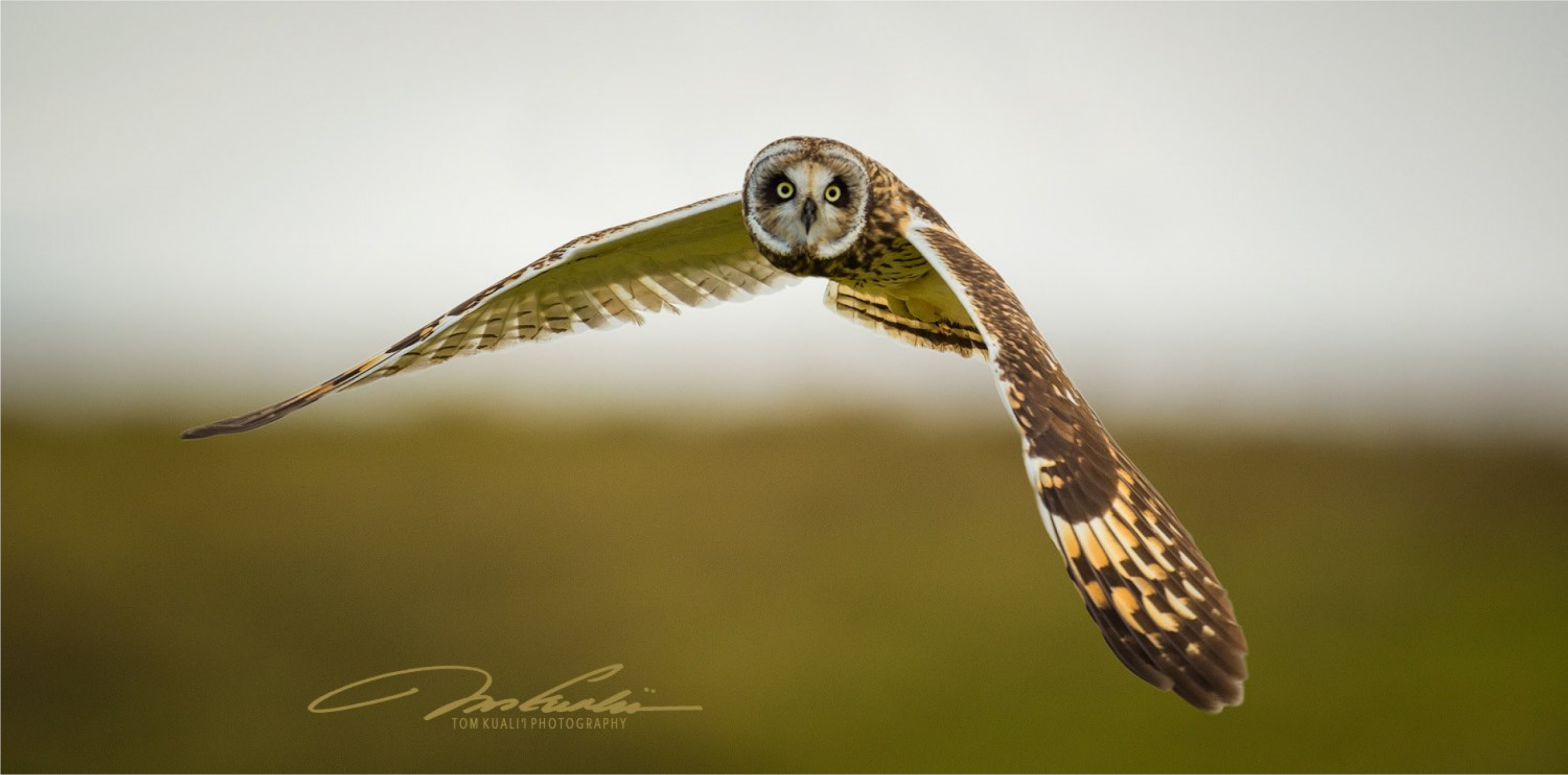
Photograph of a flying Pueo (Hawaiian Short‐eared Owl, *Asio flammeus sandwichensis*) on the Hawai'i Island, Hawai'i, USA. Photograph taken by Tom Kualii, collaborator in the Pueo project.

The Short‐eared Owl (*Asio flammeus*) is a globally distributed species present on all continents except Australia and Antarctica, with populations found in temperate, tropical, continental, and island systems (Wiggins et al., 2020). The Pueo (Hawaiian Short‐eared Owl; *Asio flammeus sandwichensis*) is an endemic subspecies of Short‐eared Owl that resides on all the main Hawaiian Islands (Figure 1). The Pueo is one of two endemic, avian, apex predators in the Hawaiian Archipelago, a role they share with the native 'Io (Hawaiian Hawk; *Buteo solitarius*; Olsen & James, 1982). A third avian apex predator, the invasive Barn Owl (*Tyto alba*), was introduced to the islands in the 1950s to control invasive rodent populations (Pratt et al., 1987). Despite suspected population declines over the last few decades that have resulted in the state‐listing of Pueo as endangered on the island of O'ahu (Mitchell et al., 2005) and recent listing by the U.S. Fish and Wildlife Service as Birds of Conservation Concern (USFWS, 2021), this subspecies remains understudied. In fact, little published information exists about the general ecology of Pueo, including their feeding habits (Mostello & Conant, 2018; Wang, 2022).

Unlike the 'Io, which is resident only on the island of Hawai'i, Pueo reside in a variety of habitat types across the islands and thus play an important ecological role. Pueo have been observed nesting and foraging in wetlands, grasslands, open scrub, agricultural lands, and high‐elevation native forests (Cotin et al., 2018; Cotin & Price, 2018; Wang, 2022) and are also seen hunting along beaches (Wilhite, 2021). Thus, Pueo co‐exist with a variety of native birds, some of which are island‐endemics and are also of conservation concern. Although continental Short‐eared Owls have been described as small‐mammal specialists, they may switch to alternative prey (e.g., birds, invertebrates) depending on seasonal variation in prey availability (Cirignoli & Podesta, 2001; Djilali et al., 2016; Holt, 1993; Srinivasulu & Srinivasulu, 2007). For instance, in North America Short‐eared Owls from coastal regions have a more variable diet and have been observed to prey upon more birds than Short‐eared Owls occupying interior regions (Holt, 1994). In Hawai'i, although our knowledge of Pueo feeding habits is still developing, the body of literature on the subject has increased in recent years (e.g., Mostello & Conant, 2018; Wang, 2022). Pueo primarily feed on rodents but appear to rely more heavily on birds and large insects than their continental counterparts. In fact, one study found that the frequency of occurrence of avian prey in Pueo pellets was as high as 69% at certain sites on the island of O'ahu (Mostello, 1996). Pueo have been documented feeding mostly on introduced avian species (Mostello & Conant, 2018, Wang, 2022), but have occasionally been observed hunting native forest passerines, as well as native seabirds and shorebirds (Mounce, 2008; Pyle, 1987; Schulmeister, 1980; Shreiber, 1967; Snetsinger et al., 1994; Tweed et al., 2006).

Here, we report novel observations of Pueo preying upon an additional native species, the Ae'o (Hawaiian Stilt; *Himantopus mexicanus knudseni*), in a wetland on the windward side of the island of O'ahu, Hawai'i. This federally endangered waterbird is a subtropical subspecies of the Black‐necked Stilt (*Himantopus mexicanus*) that breeds in wetlands across the main Hawaiian Islands (USFWS, 2011). Currently, introduced and invasive mammals (feral cats, *Felix catus*; rats, *Rattus* spp.; small Indian mongooses, *Herpestes javanicus*; dogs, *Canis domesticus*; wild pigs, *Sus scrofa*), avian predators (Barn Owls; Cattle Egrets, *Bubulcus ibis*), and amphibians (bullfrogs, *Lithobates catesbeianus*) are the main threats to Ae'o and have been confirmed consuming eggs, chicks, and/or adult Ae'o (Christensen et al., 2021; Harmon et al., 2021; USFWS, 2011). Predation by native predators has long been suspected, but to date, only a few reports have described native ‘Auku'u’ (Black‐crowned Night‐heron; *Nycticorax nycticorax*) consuming Ae'o chicks (USFWS, 2011). Here, we describe four events that provide evidence of Pueo predating Ae'o during the 2019–2021 Pueo and Ae'o breeding seasons in a wetland area on the island of O'ahu.

## 2 OBSERVATIONS

**FIGURE 2:**
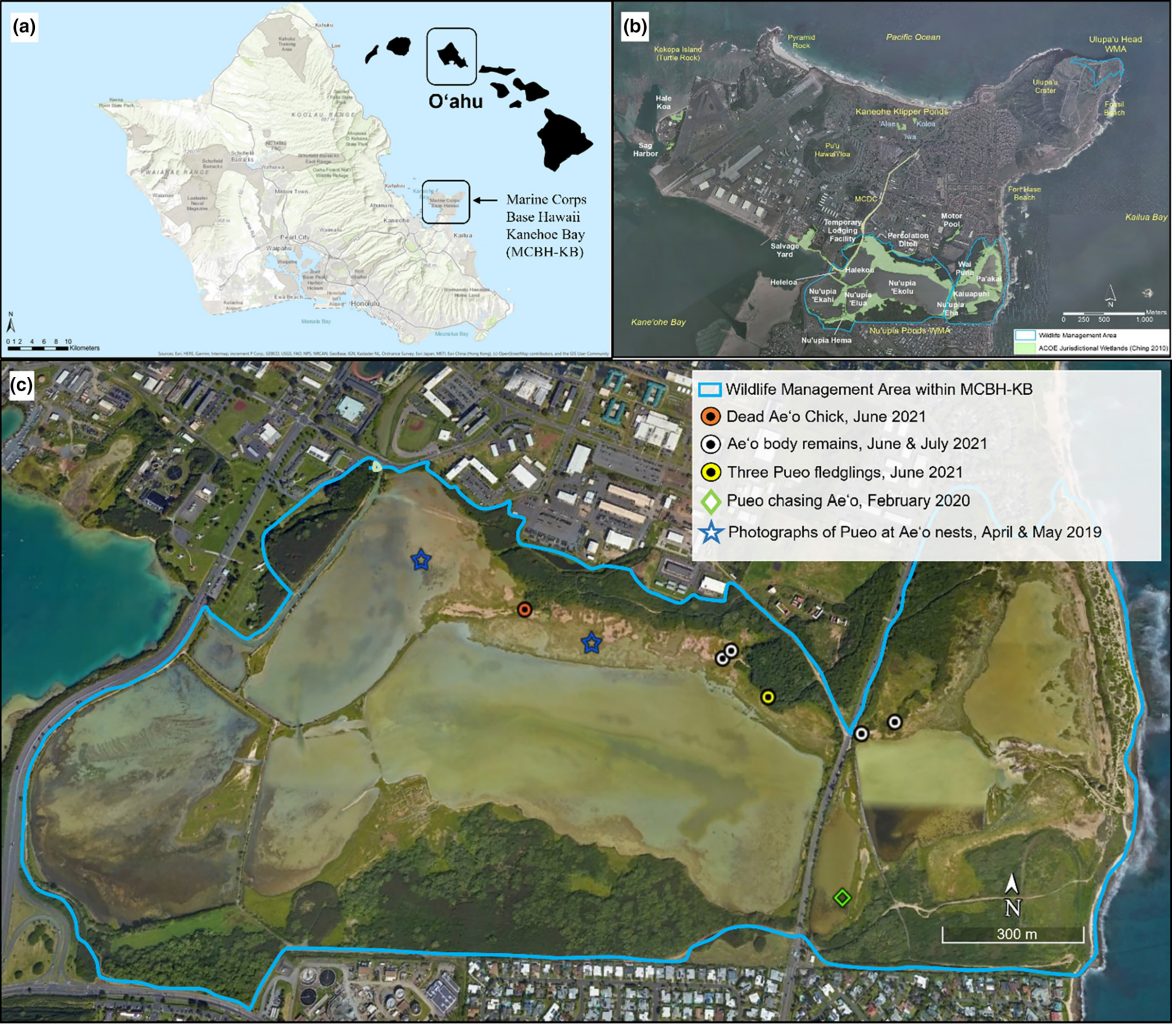
Panel (a) shows the location of the Marine Corps Base Hawaii Kaneohe Bay (MCBH‐KB), on the windward side of the island of O'ahu, Hawai'i, USA. Panel (b) shows the boundaries of our study area which was confined within the Wildlife Management Area (blue contour) of MCBH‐KB and included wetlands and ponds. This figure is adapted from the original figure used in the “Endangered Waterbird Research and Monitoring at Marine Corps Base Hawaii Kaneohe Bay” report, written by co‐authors in 2021 (Price Lab, 2021, technical report). Panel (c) shows the spatial locations of the confirmed and strongly suspected predation of Ae'o (Hawaiian Stilt, *Himantopus mexicanus knudensi*) by Pueo (Hawaiian Short‐eared Owl, *Asio flammeus sandwichensis*) in the wetland area of Marine Corps Base Hawaii Kaneohe Bay (MCBH‐KB) during the 2019–2020 and 2020–2021 breeding seasons.

Observations were conducted at the Marine Corps Base Hawaii Kaneohe Bay (MCBH‐KB) on the east side of the island of O'ahu (Figure 2). Both breeding and non‐breeding Pueo and Ae'o utilize about 60 ha of the same natural habitat (primarily composed of wetlands with mudflats and open water) at the MCBH‐KB, for nesting, resting, and foraging (Garcia‐Heras, Wang, Wilhite, Stormcrow, & Price, 2022 Harmon et al., 2021; Idle, 2023). All observations included were recorded directly by observers or through the use of motion‐activated game cameras while conducting Ae'o or Pueo surveys during the 2019–2020 and 2020–2021 breeding seasons (Figure 2).

### 2.1 Confirmed Ae'o chick predated by an adult Pueo

**FIGURE 3:**
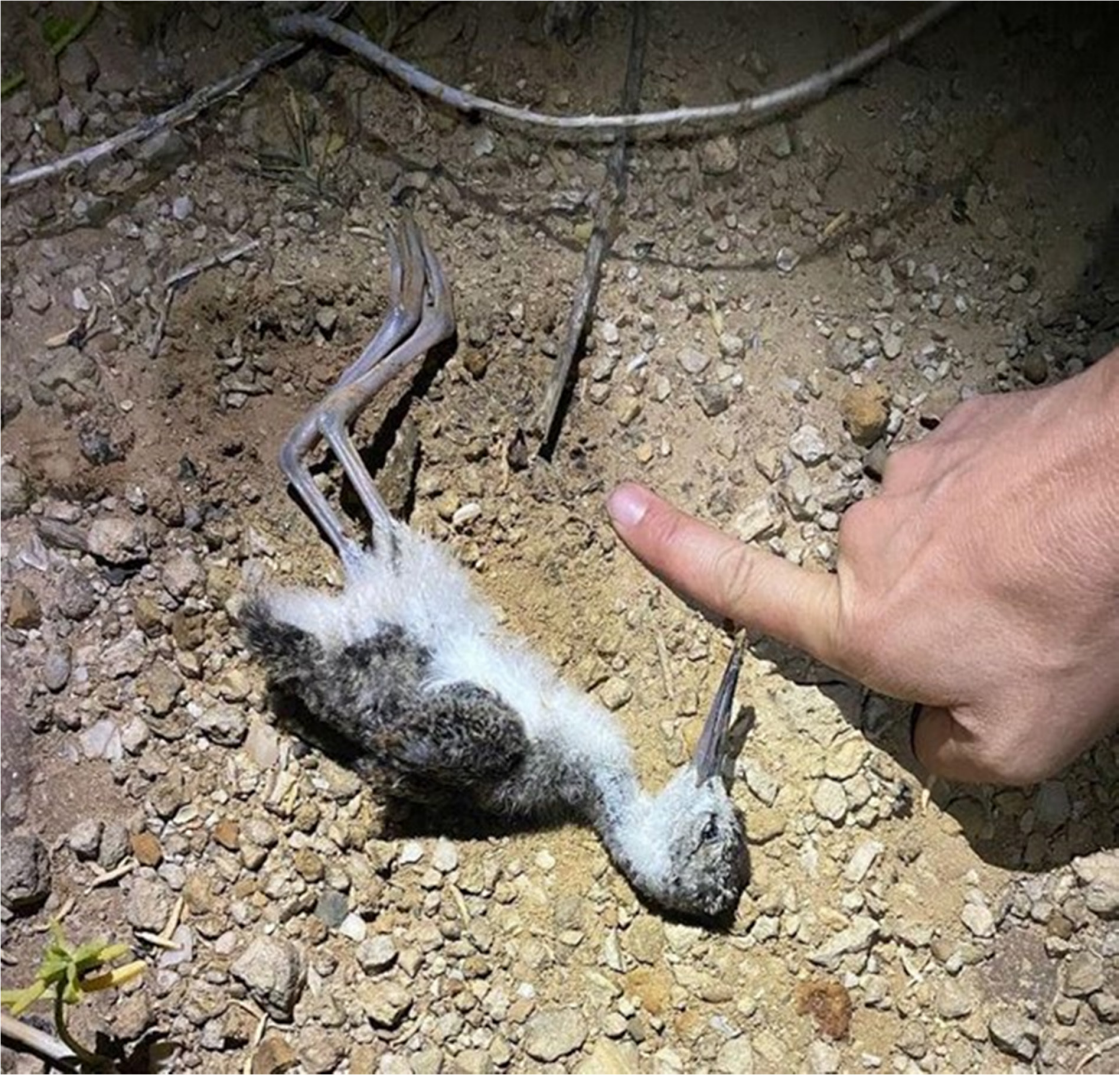
Confirmed predation of a chick Ae'o (Hawaiian Stilt, *Himantopus mexicanus knudensi*) by a Pueo (Hawaiian Short‐eared Owl, *Asio flammeus sandwichensis*) the night of June 2nd 2021, during a Pueo trapping session at Marine Corps Base Hawaii Kaneohe Bay (MCBH‐KB). Based on size and plumage, this chick was estimated to be between 2 and 3 weeks of age. Its body was still warm when found, suggesting that it was freshly caught by Pueo.

On June 2nd 2021, observers deployed a Dho Gazza net with a stuffed Pueo decoy and speaker playing Pueo calls to capture and tag Pueo. Observers began the audio playback of Pueo calls approximately 20 min before sunset. In response to the calls, several Ae'o immediately reacted defensively, flying to the area and began distraction displays, alarm calling, and mobbing the stuffed owl (Dibben‐Young et al., 2021; Harmon et al., 2021). At 20:15, after dark, an adult Pueo appeared and perched on the ground about 20–30 m away from the decoy, alarm calling at the stuffed Pueo decoy. A couple of minutes later, the Pueo flew toward the decoy and hit the Dho Gazza net, but managed to escape before observers could reach the net. Upon inspection, a dead Ae'o chick was found at the bottom of the net, dropped by the Pueo. Based on the size and plumage of the chick, it was estimated to be between 2 and 3 weeks of age (Figures 2 and 3). The body was still warm when found, suggesting that the Pueo caught it right before hitting the net. To our knowledge, this is the first documented and confirmed predation event of an individual Ae'o chick by a Pueo.

### 2.2 Suspected predation attempt: Observation of a Pueo chasing an adult Ae'o

On February 24th, 2020, while conducting Ae'o nest surveys, observers saw a Pueo roosting in the proximity of three foraging adult Ae'o. After a few minutes, the Pueo left its perch and actively chased one of the three adult Ae'o which erratically flew away, weaving and trying to evade. Both the Ae'o and Pueo, in close pursuit, flew out of view of the observers, so the result of the encounter was undetermined. On the same day, upon inspection of a second area where a different Pueo flushed from, the observers found a Pueo ground roost, indicated by a slight divot on the ground with grass pushed aside (Garcia‐Heras, Wang, Wilhite, Stormcrow, & Price, 2022); three long avian leg bones were found within the divot. Observers later identified that the leg bones were from an adult‐size Ae'o, which could have potentially been predated by Pueo (Figure 2).

### 2.3 Suspected predation events

#### 2.3.1 Body remains of several adult‐sized Ae'o

**FIGURE 4:**
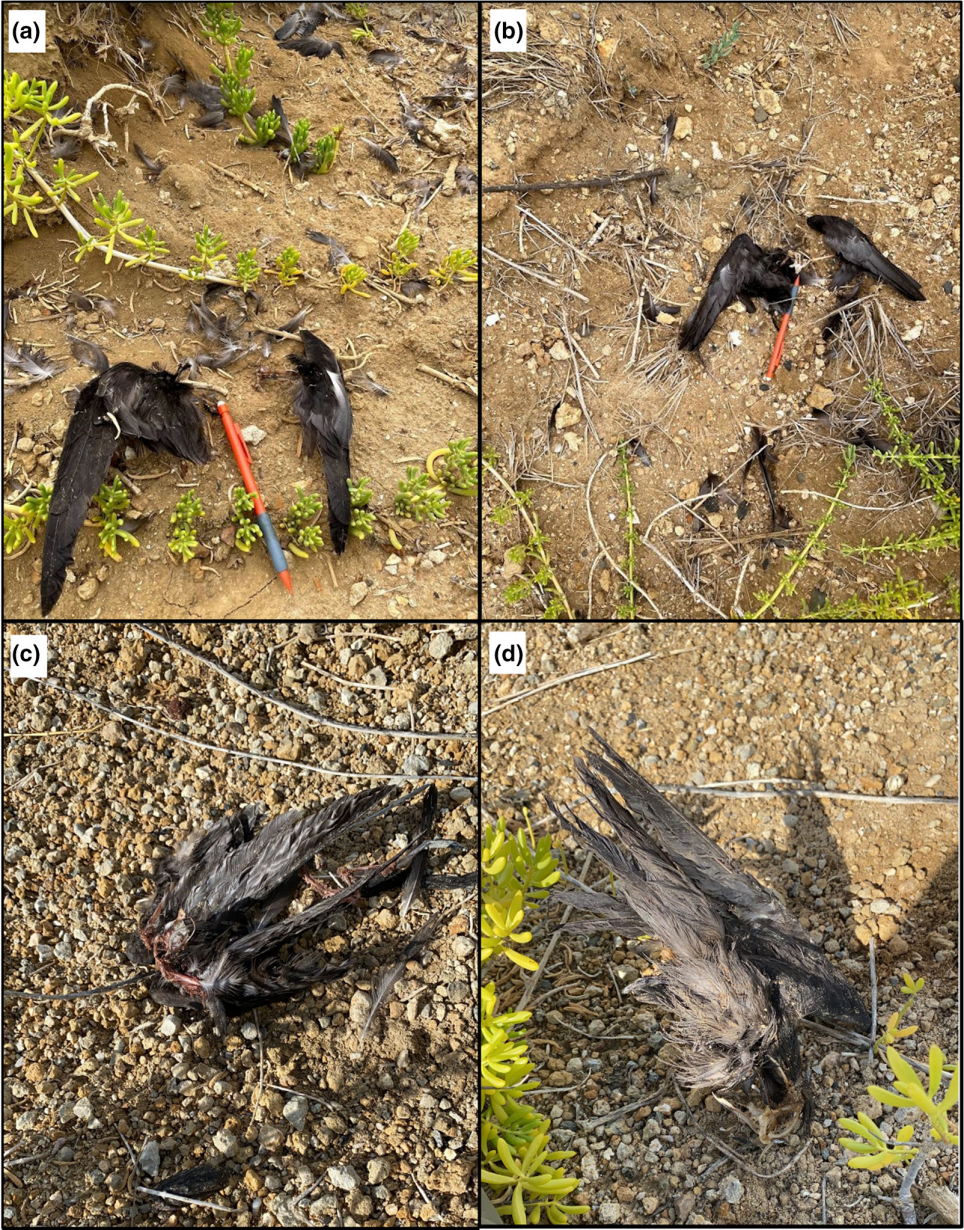
Four of the 10 body remains of adult‐sized Ae'o (Hawaiian Stilt, *Himantopus mexicanus knudensi*) found by team members within the wetland area of Marine Corps Base Hawaii Kaneohe Bay (MCBH‐KB) during June and July 2021. The exact age of the birds could not be determined as the plumage was not always intact or identifiable: birds on panel (a) and (b) seem to have recently been killed as the plumage coloration is not yet sun‐bleached, while the remains in panels (c) and (d) seem to be older (sun‐bleached).

From June to July 2021, observers found the remains of ten adult‐sized Ae'o all within an approximately 350 m radius of each other (Figures 2 and 4). Carcasses ranged from old to fresh; age of the carcass was determined by how sun‐bleached the plumages were. The exact age of the Ae'o predated could not be determined as the plumage was not always intact or identifiable. Upon closer inspection of the Ae'o remains, we found that the wings were still attached to the keel bone via either the furcula or the coracoid, the plucked feathers lying around the carcasses were removed from the body prior to the flesh being picked from the bones with no damages to the feather's calamus, and the remaining bones were unbroken—all clear signs of predation by a raptor. For comparisons to other potential Ae'o predators in the area, feral cats characteristically bite and shear feathers, dogs consume the entire body and crush bones, and bull frogs are too small to consume full‐grown Ae'o.

Furthermore, these remains were all found within an approximately 190 m radius from the location where three juvenile Pueo and both their parents were repeatedly observed throughout June and July, and about 600 m from where we attempted to trap the adult Pueo mentioned in the previous observation. Based on both the condition of the carcasses and the proximity of the carcasses to known Pueo breeding and hunting areas, we determined that these Ae'o were consumed by an owl species, and very likely by breeding Pueo instead of a Barn Owl.

#### 2.3.2 Photo of Pueo at two Ae'o nests

**FIGURE 5:**
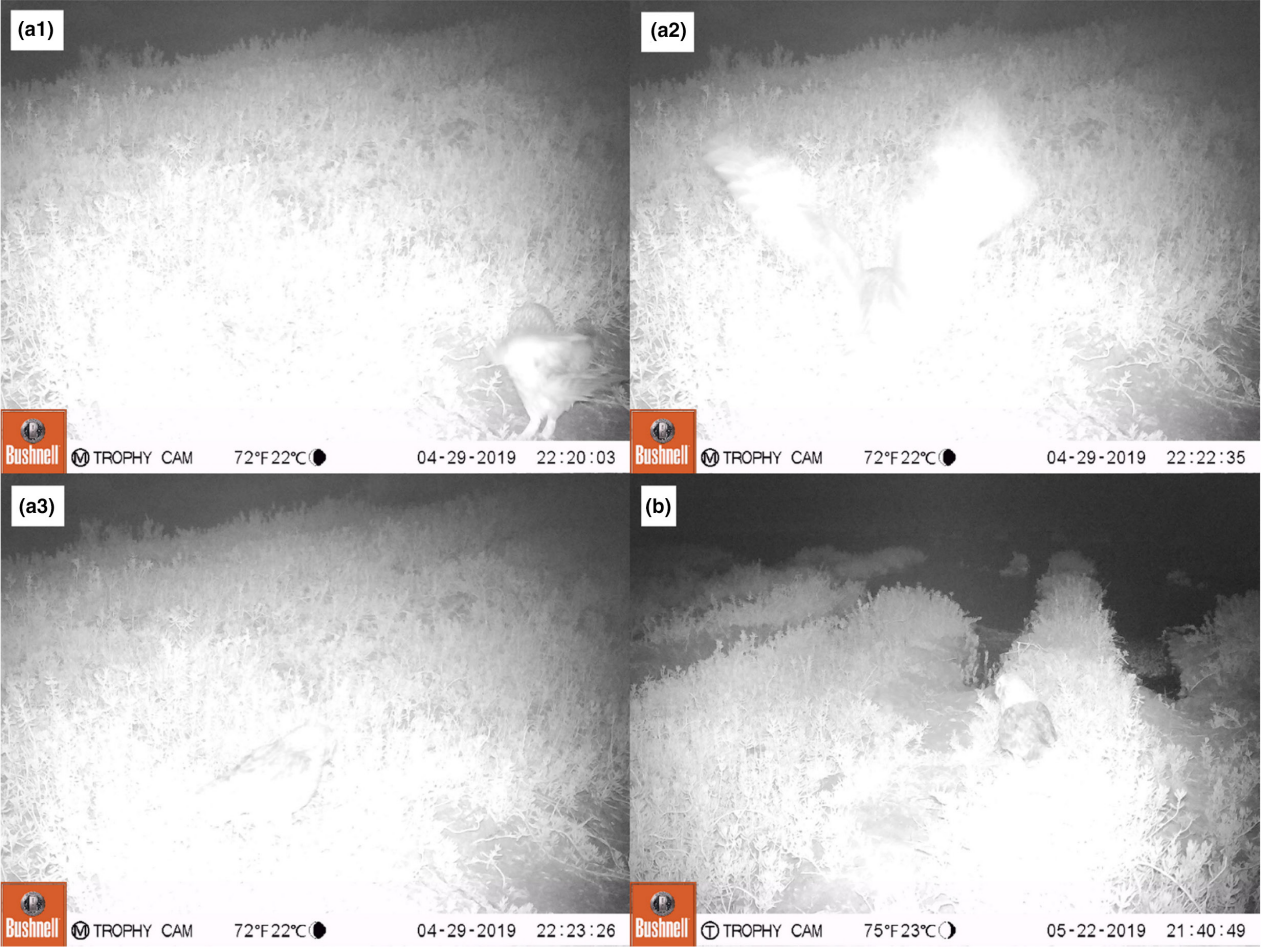
Selection of four pictures taken by a motion‐activated game camera pointed at two active Ae'o (Hawaiian Stilt, *Himantopus mexicanus knudensi*) nests that were triggered by a Pueo (Hawaiian Short‐eared Owl, *Asio flammeus sandwichensis*) visiting the nests: (a1–a3) during the night of April 29th, 2019 for the first nest, and (b) during the night of May 22nd 2019 for the second nest. Both Ae'o nests had successfully hatched chicks shortly prior to the series of photos being captured.

During the 2019 Ae'o nesting season, 45 motion‐activated game cameras were deployed at active Ae'o nests to capture nest fate (Price Lab, 2021, technical report). Among those 45, two nest cameras were motion‐triggered by a Pueo standing at or near an Ae'o nest (Figure 5). The first Ae'o nest visited by a Pueo had successfully hatched chicks shortly before the series of photos were captured, during the night of April 29th, 2019 between 22:20 and 22:50. The second Ae'o nest visited by a Pueo occurred on May 22nd, 2019 at 21:40, after three of the four Ae'o eggs had hatched. Right after hatching, Ae'o chicks disperse from the nest but are thought to remain in close proximity until all the eggs hatch (Coleman, 1981). Thus, a predator at the nest during this one‐to‐two‐day period post‐hatch would likely encounter young chicks in the immediate surroundings of the nest. Although the survival of the Ae'o chicks was undetermined, the presence of the Pueo at the nests and its surroundings so soon after hatching provides further evidence that Pueo predate, at least occasionally, Ae'o chicks.

## 3 DISCUSSION

Although our knowledge about the diet composition and feeding habits of Pueo in the Hawaiian Islands is still developing, evidence suggests that this subspecies has a more variable diet than continental populations of Short‐eared Owls (Clark, 1975), with a higher consumption of birds (Mostello & Conant, 2018; Wang, 2022). Predation of waterbirds by Pueo has been suspected, but prior to this study was not confirmed nor published. Here, we provide evidence confirming that Pueo do prey upon Ae'o chicks and that they very likely also prey upon Ae'o adults, potentially in greater frequency than previously imagined. To our knowledge, these observations are the first published accounts of predator–prey interactions between these two subspecies, and more globally between these two widely distributed species, as Short‐eared Owls and various species of Stilts likely occupy similar niches in wetlands around the world.

Although we did not directly observe Pueo predating adult Ae'o and many potential predators of adult Ae'o are present within MCBH‐KB, the condition of the Ae'o remains point toward raptor predation rather than mammalian predation. At least one Barn Owl was present in the wetland area during the study period, a species reported to prey upon seabirds and waterbirds across the Hawaiian Archipelago (Byrd & Telfer, 1980; Raine et al., 2019; Underwood et al., 2014; VanderWerf et al., 2007). While we cannot reliably distinguish between predation caused by Barn Owls versus Pueo based on the conditions of the remains alone, the proximity of the Ae'o remains to both a Pueo breeding territory with three chicks and to the location where we confirmed an adult Pueo predating upon Ae'o chick, leads us to believe that the predation of the adult‐sized Ae'o were due to Pueo. Furthermore, we frequently observed Ae'o reacting defensively to the Pueo calls and decoy that we used during Pueo trapping sessions, with similar reactions each time we saw a Pueo flying over areas where nesting Ae'o were present, day or night. Altogether, this suggests that Pueo are indeed perceived as a threat by adult Ae'o, and that Ae'o may form a more important component of the Pueo diet at the MCBH‐KB site than we previously had anticipated.

Overall, birds are an important component of Pueo diet and predation rates of birds may be even higher than previous studies have found, above all for Pueo occupying wetland ecosystems such as MCBH‐KB. In fact, recent analyses from pellets collected at previously identified Pueo roost sites, from both breeding and non‐breeding Pueo, showed that at the MCBH‐KB site, the proportion of birds in Pueo diet was slightly greater than the proportion of rodents (Garcia‐Heras, Wang, Wilhite, Stormcrow, & Price, 2022). In comparison, pellet analyses collected at an open‐grassland site on the west side of O'ahu during the same period of time showed that rodents were a greater proportion of Pueo diet than birds (Garcia‐Heras, Wang, Wilhite, & Price, 2022; Wang, 2022). Overall, none of the MCBH‐KB samples seemed to contain Ae'o remains. This was, however, not too surprising as raptor pellet analyses are strongly biased toward overrepresenting mammalian prey and underrepresenting avian prey and other smaller prey such as reptiles or insects (Garcia‐Heras, Mougeot, Arroyo, et al., 2017; Garcia‐Heras, Mougeot, Simmons, & Arroyo, 2017; Marchesi et al., 2002; Simmons et al., 1991). Direct observations of predation events thus remains important in fully understanding a species' diet (Lewis et al., 2004; Redpath et al., 2001).

Our observations highlight an important conservation challenge, in which a native predator of conservation concern is preying upon another native and endangered bird species, compounding mortality due to other causes such as introduced predators. Thus, invasive predator removal operations are critical to preventing the loss of native Hawaiian birds but could also be crucial in allowing native predator–prey interactions to be restored (Underwood et al., 2014; Young et al., 2013). Further, native species such as Pueo that are culturally important for Native Hawaiians as ‘aumakua’ (deified ancestors or ancestral guardians; Loebel‐Fried, 2002, Stormcrow, 2023) may occasionally predate other endangered or threatened birds. While predation by native predators should be appropriately accounted for in population modeling and management plans for endangered prey species, removal of native predators is unlikely to be appropriate, and instead, these interactions should be viewed as a potential sign of a recovering native ecosystem. Further investigation is needed to determine whether these predation events occur sporadically or if they are indeed a common occurrence across the Archipelago, as this may have potential impacts on the breeding success and overall survival of Ae'o when compounded with predation by both native and non‐native predators, and to the concurrent conservation of both subspecies in Hawai'i.

## AUTHOR CONTRIBUTIONS

**Marie‐Sophie Garcia‐Heras:** Conceptualization (equal); data curation (equal); investigation (equal); visualization (equal); writing – original draft (lead); writing – review and editing (lead). **Jessica L. Idle:** Conceptualization (equal); data curation (equal); investigation (equal); visualization (equal); writing – review and editing (equal). **Olivia Wang:** Conceptualization (equal); data curation (supporting); investigation (equal); visualization (equal); writing – review and editing (equal). **Kristen C. Harmon:** Conceptualization (equal); data curation (equal); investigation (equal); visualization (equal); writing – review and editing (equal). **Chad J. Wilhite:** Conceptualization (equal); data curation (equal); investigation (equal); visualization (equal); writing – review and editing (equal). **Kaleiheana‐a‐Pōhaku Stormcrow:** Conceptualization (supporting); writing – review and editing (equal). **Lesley N. Davidson:** Data curation (equal); writing – review and editing (supporting). **Wade H. Naguwa:** Data curation (equal). **Lauren S. Katayama:** Data curation (equal). **Melissa R. Price:** Conceptualization (equal); funding acquisition (lead); investigation (equal); project administration (lead); visualization (equal); writing – review and editing (equal).

## CONFLICT OF INTEREST STATEMENT

The authors declare no conflicts of interest.

## Data Availability

Following the instructions of WOA Ecology and Evolution Editorial Assistant, we have now provided some of the respective data as a supporting information under “Additional File for Review but NOT for publication.” However, as our observations were collected within the military base of Marine Corps Base Hawaii Kaneohe Bay (MCBH‐KB), Hawai'i, USA, we are unable to share some sensitive information, such as the GPS locations of the events. Thus, additional data that support the observations of this study will only be available upon request.
